# Viral hepatitis and liver cancer

**DOI:** 10.1098/rstb.2016.0274

**Published:** 2017-09-11

**Authors:** Marc Ringehan, Jane A. McKeating, Ulrike Protzer

**Affiliations:** 1 Institute of Virology, Technical University of Munich/Helmholtz Zentrum München, Trogerstrasse 30, 81675 Muenchen, Germany; 2 Department of Internal Medicine II, University Hopsital rechts der Isar, Technical University of Munich, Ismaninger Strasse 22, 81675 Muenchen, Germany; 3 German Center for Infection Research (DZIF), partner site Munich; 4 Institute for Advanced Science, Technical University of Munich, Muenchen, Germany; 5 Nuffield Department of Medicine, University of Oxford, Oxford, UK

**Keywords:** hepatitis B, hepatitis C, chronic viral hepatitis, hepatocellular carcinoma, hepatocarcinogenesis, virus-induced cancer

## Abstract

Hepatitis B and C viruses are a global health problem causing acute and chronic infections that can lead to liver cirrhosis and hepatocellular carcinoma (HCC). These infections are the leading cause for HCC worldwide and are associated with significant mortality, accounting for more than 1.3 million deaths per year. Owing to its high incidence and resistance to treatment, liver cancer is the second leading cause of cancer-related death worldwide, with HCC representing approximately 90% of all primary liver cancer cases. The majority of viral-associated HCC cases develop in subjects with liver cirrhosis; however, hepatitis B virus infection can promote HCC development without prior end-stage liver disease. Thus, understanding the role of hepatitis B and C viral infections in HCC development is essential for the future design of treatments and therapies for this cancer. In this review, we summarize the current knowledge on hepatitis B and C virus hepatocarcinogenesis and highlight direct and indirect risk factors.

This article is part of the themed issue ‘Human oncogenic viruses’.

## 1. Hepatitis B virus infection

Hepatitis B virus (HBV) is one of the most common chronic infections worldwide, with an estimated 257 million chronically infected subjects, and the leading cause for hepatocellular carcinoma (HCC) worldwide [1]. Owing to the high risk of developing end-stage liver disease or HCC, chronic hepatitis B (CHB) is associated with high mortality (15–40% in 10–25 years) [2], with about 880 000 deaths per year due to complications of CHB (WHO 2017). The occurrence of symptoms in the context of acute infection is age-dependent. Most infections in children are clinically silent. In adults, up to 70% of cases show subclinical hepatitis with an increase in transaminases, and in up to 30% of cases a transient jaundice and flu-like prodromal stage [1]. Acute HBV infection can also result in fulminant hepatitis with liver failure (less than 1% of cases); however, acute symptoms are usually transient and self-limiting. The clinical course of CHB is often inapparent until late-stage liver disease is evident.

The prevalence of HBV infections varies in different geographical regions, with highest rates in sub-Saharan Africa and East Asia, where 5–10% of the adult population is chronically infected. High rates of infection are reported in the Amazon and southern parts of eastern and central Europe. In the Middle East and the Indian subcontinent, an estimated 2–5% of the population is chronically infected. Less than 1% of the Western European and North American population is chronically infected (WHO 2016). This risk of acquiring HBV infection was drastically reduced by increased hygiene standards, screening of blood products and introduction of a prophylactic vaccine [3]. Despite the availability of this vaccine for more than 40 years, the number of infections remains high, owing in part to the failure to implement vaccination programmes and also to a high number of perinatal infections in endemic areas [1,3]. To reduce perinatal infection, nucelos(t)ide analogue treatment of highly viraemic mothers may be necessary, in addition to postnatal treatment with hepatitis B immunoglobulin and HBV vaccination [4]. Despite these treatments, more than 10% of infants born to highly viraemic mothers acquire HBV infection despite active and passive vaccination [4–6]. Lamivudine, telbivudine and tenofovir have been shown to be safe and to reduce the risk of intrauterine and perinatal HBV transmission when given in concert with passive and active vaccination [4,6].

HBV is a partially double-stranded DNA virus that replicates via reverse transcription. In contrast with retroviruses, such as human immunodeficiency virus (HIV), integration of the viral DNA is not an essential step in the virus life cycle. HBV is characterized by its narrow host range and tissue tropism to replicate in hepatocytes. The virus persists via an episomal transcription template within the nucleus of infected hepatocytes that is defined as covalently closed circular DNA (cccDNA) [7]. The viral genome has four overlapping open reading frames encoding the structural core (HBc) and envelope proteins, the viral polymerase/reverse transcriptase and regulatory X protein (HBx), which is regarded as an oncoprotein. Three envelope proteins of different sizes (small (S), medium (M) and large (L)) are encoded by the same open reading frame with M and L carrying N-terminal extensions. Although regarded to be irrelevant for the virus life cycle, non-circularized HBV genomes have been reported to integrate into the hepatocellular genome [8].

A limited number of HBV virions (1–10) are sufficient to initiate infection, and the virus is transmitted via contact with blood or body fluids during sexual intercourse and vertically from mother to child. The latter accounts for the high number of chronic carriers because infection around birth and during early childhood results in high chronicity rates of greater than 90%. By contrast, infection of adolescents or adults largely results in acute infections with only 1–5% of subjects developing chronic infection [1]. The ‘natural’ history of chronic HBV infection is classified in specific stages that are defined by hepatic inflammatory activity and viral replication rates [9]. High viraemia is associated with the expression of pre-core antigen (HBeAg), while anti-HBe serum reactivity is observed in low-replicating infection or when viral mutants emerge. Traditionally, the HBeAg status has been used as a parameter to assess viral replicative fitness and disease prognosis [2]. However, current studies support the predictive value of HBV-DNA levels to estimate HCC risk and disease prognosis [9].

## 2. Hepatitis C virus infection

Worldwide, 140 million infections with hepatitis C virus (HCV) are estimated [10,11]. The lack of proof-reading capacity of the HCV-encoded polymerase along with high replication rates results in a high mutation rate and genesis of a heterogeneous but closely related quasi-species [12]. HCV is transmitted via parenteral routes, occurs in industrialized countries via intravenous drug abuse or by invasive sexual practices and is rarely transmitted from mother to child. Transmission has been limited by improving hygienic standards. In contrast with HBV, the risk of viral persistence and the development of chronic HCV infection in children are lower than those in adults. HCV has a very different prevalence depending on demographic factors: approximately 1.6% in the USA, less than 0.5% in Northern Europe and up to 3% in rural regions of Romania [11]; the most-affected regions are Central and East Asia and North Africa.

Acute HCV infection is asymptomatic in most cases, and only 15% of cases are symptomatic with symptoms such as fatigue, nausea, joint pain or signs of liver damage (jaundice and increased liver enzymes). The majority of adults develop chronic infection (55–85%), with 15–45% resolving infection within the first six months. It has been reported that 350 000–500 000 people die each year from HCV-related liver diseases such as liver cirrhosis or HCC (WHO 2016). Chronic hepatitis C (CHC) shows a variable clinical course, ranging from mild histopathological changes to highly active hepatitis and the development of liver fibrosis, cirrhosis and HCC over several decades.

CHC is a slowly progressive disease characterized by persistent hepatic inflammation resulting in liver fibrosis and liver cirrhosis. Since fibrosis progression is not linear, estimating its prognosis is difficult. Persistent hepatic inflammation leads to the development of cirrhosis in approximately 10–20% of patients over 20 years, while other studies report a 40% cirrhosis risk over 30 years [11,13]. Once high-grade fibrosis (Ishak grade 3 or 4) or cirrhosis has developed, there is a 1–5% annual risk of developing HCC. However, only a minority of HCV-infected individuals develop cancer, suggesting a complex interplay between viral gene expression and host and environmental factors to promote hepatocyte transformation and carcinogenesis. Transgenic mice engineered to express the HCV genome show an increased risk for HCC [14]; however, the lack of small animal models supporting HCV infection and associated pathologies limits our understanding of pathways underlying HCV-associated HCC.

HCV is a single-stranded, positive-sense RNA virus that encodes a single polyprotein that is post-translationally cleaved into structural (S) and non-structural (NS) proteins. Structural proteins include core protein, envelope E1 and E2 glycoproteins and p7 protein, and constitute the viral particle. Non-structural proteins (NS1, NS2, NS3, NS4A/B and NS5A/B) support viral genome replication and particle assembly. HCV replicates in the cytoplasm of hepatocytes and is unique among cancer-causing viruses in not encoding oncoproteins or integrating its genome into the host chromosomal DNA. The mechanisms underlying HCV-associated carcinogenesis are mainly indirect effects of virus de-regulating host cellular processes, including (i) increased hepatocyte proliferation and steatosis, (ii) virus-induced inflammation and oxidative stress inducing genomic mutations and genome instability, (iii) mitochondrial damage and induction of reactive oxygen species (ROS) and (iv) effects of virus-induced host immune responses.

## 3. Hepatitis delta virus infection

The hepatitis delta virus (HDV) is a satellite virus that depends on HBV for generation of progeny virus and propagation. The HDV genome comprises a circular single-stranded RNA of around 1700 bases. The antigenomic open reading frame encodes the only viral protein, hepatitis delta antigen (HDAg), that exists in two forms, the small- and the large-HDAg, and HDV particle assembly is dependent on the HBV envelope glycoprotein. Thus, HDV can only establish infection in the presence of HBV co-infection. HDV infects 15–20 million subjects worldwide and causes the most severe form of viral hepatitis. Several studies (reviewed in [15]) have shown that chronic HDV co-infection leads to a more pronounced inflammation and severe liver disease than HBV mono-infection. In addition, HDV accelerates the course of progression to fibrosis and cirrhosis, and increases the risk for HCC development and early decompensation of cirrhosis [16]. HDV accounts for almost half of all cases of cirrhosis and HCC in high-epidemic regions such as southeast Turkey, Italy or Mongolia. To date, no specific antiviral treatment is available for HDV.

HDV infection is not cytopathic and HDAg is not directly oncogenic, but high-level expression and nuclear translocation can activate NFκB- and STAT3-mediated inflammatory response and oxidative stress, which promote HBV oncogenesis [17]. HDV infection is characterized by a markedly increased inflammatory liver disease with necro-inflammation and increased hepatocyte turn-over compared with HBV mono-infection, rendering active hepatitis as the lead course why HDV accelerates HCC development.

## 4. Hepatocellular carcinoma epidemiology, risk factors and treatment options

Worldwide, liver cancer is the second leading cause of cancer-related death in men, with 745 000 deaths per year, and the sixth most common cancer, with rising incidence (approx. 800 000 new cases each year) [18]. HCC represents approximately 90% of all primary liver cancer cases, shows a clear gender disparity towards males and is a major cancer in less developed regions, with a correlation to HBV surface antigen prevalence. Chronic HBV and HCV infections represent the leading cause for HCC (60–70%), with a total incidence of 16/100 000 globally. In most of Africa and Asia, HBV is the single leading risk factor for HCC, whereas in Japan, northern Europe and the USA HCV is the major risk factor [19]. The risk of developing HCC is 10- to 25-fold higher in CHB [20] compared with non-infected controls, and up to 17-fold increased in HCV-associated liver cirrhosis [19]. While HCC in HCV infection rarely occurs without liver cirrhosis, CHB without any obvious liver inflammation *per se* confers a risk for HCC development. The highest risk for HCC development is associated with co-infection of HBV with HDV, HCV or HIV.

A reduction of HCC incidence in some high-risk countries can be attributed to HBV vaccination programmes and increased hygienic standards mainly because aflatoxins are known to increase HCC risk [18,21]. This is as well mirrored by HCC attributable to HBV being far less common in northwestern Europe (18%) and the USA (20% of cases) when compared with 51% in eastern/southern Europe and 65% in the Far East and China [21,22]. Nevertheless, liver cancer incidence steadily increased in areas with historically low rates, including parts of Oceania, Western Europe and North America. For example, a rise from 2.6 to 8.6 per 100 000 was observed in the USA between 1975 and 2011, which was partially attributable to the increase in HCV-associated liver cirrhosis 20–40 years after infection and high prevalence of metabolic syndrome as an independent risk and cofactor [21].

HCC high mortality is most likely due to the resistance of this tumour to chemotherapy along with concomitant complications of end-stage liver disease and frequent diagnosis at late stages when limited treatment options are available. Thus, in contrast with other cancer types, HCC classification is not based on the ‘classical’ TNM tumour-staging/grading but on a clinical score based on the number of HCC nodules, size, vascular invasion, stage of cirrhosis and the Eastern Cooperative Oncology Group Performance Status: the Barcelona Clinic Liver Cancer (BCLC) staging classification [23–25]. While in the early stages (BCLC 0/A) patients are eligible for potentially curative therapies (i.e. surgical resection and liver transplantation (within MILAN criteria [26]) and radiofrequency ablation and median survival rates of 60 months and beyond can be reached. However, fewer than 40% of patients are diagnosed at early stages, and in advanced HCC only palliative treatment options are available, with poor overall survival [23–25].

Thus, there is an urgent need for effective and tolerable treatments for HCC. However, besides the multikinase inhibitors sorafenib and regarofenib, which improve median overall survival by only approximately three months [27], more than 100 trials evaluating chemotherapy or targeted therapies in HCC failed to show survival advantages [28,29]. New promising approaches include immune checkpoint inhibitors [30] and adoptive T-cell transfer approaches [31]. The poor outcome of targeted therapies in late-stage HCC is a result of a diverse spectrum of HCC subtypes, without common growth addiction loops. Thus, it is of the utmost importance to understand the causes of HCC development and to find novel approaches.

Substantial progress has been made in understanding the molecular mechanisms of hepatocarcinogenesis. In chronic viral hepatitis as well as in other aetiologies of HCC (i.e. alcoholic liver disease, non-alcoholic fatty liver disease and certain rare metabolic, autoimmune or hereditary liver diseases) chronic inflammation, cell death and compensatory hepatocyte proliferation referred to as necro-inflammation is an important driver of liver fibrosis. The single most evident risk factor for HCC is liver cirrhosis. Well-known cofactors for HCC development are increasing age (greater than 40 years), duration of infection, male gender, alcohol consumption, cigarette smoking, co-infection with HBV/HCV, HDV or HIV and exposure to aflatoxin B1 [32].

The risk for HCC in CHB and CHC is closely linked to liver inflammation during chronic infection. Both viral infections are non-cytopathic and liver damage is thought to be induced by viral-specific CD8^+^ T- and natural killer (NK) cells rather than by the viruses themselves [33,34]. Events driving hepatocyte transformation include DNA damage, epigenetic modifications, mitochondrial alteration, senescence and chromosomal aberrations [35]. ROS or nitrogen compounds are produced by macrophages and neutrophils in inflammation, which can attack DNA, leading to adducts that impair base-pairing and/or block DNA replication and transcription, and to base loss or DNA-strand breaks [36].

## 5. Clinical features of hepatitis B virus-associated hepatocellular carcinoma

While in CHC HCC almost exclusively develops in liver cirrhosis, up to 20% of HBV-driven HCC cases occur in the absence of cirrhosis [27,37]. The levels of HBV replication reflected by HBV-DNA serum titres, concomitant liver inflammation and necroinflammatory tissue damage have been confirmed as the most important predictors of disease progression and HCC development. The risk for HCC correlates with HBV viraemia [38]. This was first described in the REVEAL-HBV study, where mortality increased with baseline HBV-DNA levels from 9 (fewer than 300 copies ml^−1^) to 267 (more than 10^6^ copies ml^−1^) deaths due to chronic liver disease and cirrhosis, and 73–816 deaths per 100 000 person-years due to HCC, respectively [38]. Multivariate Cox regression analyses of risk factors predicting progression to mortality identified increasing HBV-DNA levels as the strongest independent predictor of death from chronic liver disease and cirrhosis, and this was second to cirrhosis in predicting death from HCC [39]. This effect was specific because there was no association between serum HBV-DNA levels and non-liver-related mortality.

A study with 2946 HBsAg seropositive individuals during the natural course of disease showed a reduced risk of developing HCC after seroclearance of HBeAg and in particular after resolving HBV-DNA and HBsAg expression during follow-up [40]. Among HBeAg seronegative participants with detectable serum HBV-DNA at study entry, the lifetime cumulative incidence of HCC was 14.2% if patients remained HBV-DNA and HBsAg positive, 6.6% after clearance of HBV-DNA without loss of HBsAg and 4.0% even after seroconversion to anti-HBs [40]. Importantly, patients cured of CHB remain at risk of developing HCC.

## 6. The role of hepatitis B virus in promoting hepatocarcinogenesis

CHB-associated inflammation and liver damage foster the accumulation of genetic and epigenetic defects that lead to the onset of HCC. However, a direct and specific contribution of the virus is supported by clinical observations and experimental data. Thus, HCC develops in 10–20% of HBV-infected individuals who lack any sign of cirrhosis. HCC can even develop in the absence of inflammation, which is in stark contrast with most other aetiologies associated with HCC [20]. HBV has a number of features that are known to contribute to HCC development independently of inflammation [11,41,42]. HBV genomes can integrate into the host genome and induce chromosomal alterations and insertional mutagenesis of cancer genes [41]. High-throughput next-generation sequencing approaches identified some recurrent sites for integration in biopsies taken from HCC but at low incidence (i.e. telomerase reverse transcriptase (TERT), myeloid/lymphoid or mixed-lineage leukemia 4 (MLL4), cyclin E1 (CCNE1), neurotrophic tyrosine kinase receptor type 2 (NTRK2), interleukin-1 receptor-associated kinase-like 2 (IRAK2), mitogen-activated protein kinase 1 (MAPK1)) [43,44]. In addition, viral promoter-driven human transcripts have been reported within or close to repetitive, non-coding sequences, such as LINEs (long interspersed nuclear elements) or SINEs (short interspersed nuclear elements) [45]. Although, taken together, HBV integration is random and rarely leads to direct oncogene activation or inactivation of a common tumour suppressor [46], it is widely accepted that integration contributes to the genetic instability of the hepatocyte and marks clonally growing hepatocytes.

Hepatocytes are self-renewing cells [47] that can proliferate to maintain liver mass during injury [48]. Necroinflammatory viral hepatitis is associated with increased hepatocyte proliferation that can maintain integrated HBV-DNA, and consequently, epigenetic and genetic dysregulation, including damage via HBV-DNA integration, will increase over time. Mason *et al.* [49] reported that random HBV integration events increased hepatocyte turn-over and that clonal expansion of hepatocytes occurs in HBV-infected individuals before liver damage is clinically apparent. Since integration of HBV-DNA is a risk factor for HCC development, this study proposes a model where HBV-associated hepatocarcinogenesis occurs prior to the onset of liver fibrosis or cirrhosis and provides an explanation for the limited efficacy of antiviral therapies to limit HCC progression when initiated late in the course of disease.

HBV particles package an incomplete partially double-stranded circular DNA that is imported into the nucleus where it is ‘repaired’ by cellular enzymes to cccDNA. The incomplete DNA is recognized in the nucleus as damaged and induces a DNA damage response [7,50]. While the DNA damage response can activate cell cycle checkpoints and DNA repair pathways that counteract genomic mutations in cancer, it can also lead to histone degradation, which enhances chromatin dynamics and recombination rates [51] and may promote genomic instability in HBV-associated HCC.

The HBV-encoded envelope and HBx proteins are reported to directly contribute to hepatocyte transformation via distinct and non-overlapping pathways. The envelope proteins can induce endoplasmic reticulum stress via an unfolded protein response, and transgenic mice engineered to express the envelope proteins develop liver cancer [42]. HBV-DNA sequences coding for a C-terminally truncated envelope protein are frequently found integrated in HCC. This truncated M protein may increase hepatocyte proliferation, trigger activation of c-Raf-1/Erk2, Ap-1 and NF-κB pathways and show *trans*-activation potential [52].

HBx plays a role in hepatocyte transformation and is a driver of HCC progression. HBx is usually expressed at low levels during infection. With increasing integration frequency of HBV-DNA during infection and associated increase in hepatocyte proliferation, relative HBx expression levels can increase and transcripts are frequently detected at high levels in HBV-related HCC [53]. HBx regulates expression of a plethora of genes involved in signal transduction pathways, cell cycle control, metastasis, transcriptional regulation, immune response and metabolism, and has been implicated as having a direct oncogenic function (summarized in [46]). Changes in signalling cascades and cellular integrity may occur from increased cytosolic calcium levels through HBx interference which stimulates HBV replication, but may have oncogenic potential by activating Src- and Ras-signalling [54]. However, the physiological relevance of these findings is difficult to prove where low-level expression of viral proteins, including HBx in the infected liver, precludes confirmation of *in vitro* and *in vivo* mouse studies which frequently overexpress HBx.

HBx is essential for HBV transcription from cccDNA and for initiating and maintaining virus replication [55]. HBx activation of HBV transcription has been proposed to be linked to chromatin modulation because HBx association with cccDNA correlates with the recruitment of acetyltransferases CBP/P300 or PCAF and acetylation of histone H3 [56]. HBx has also been reported to inhibit the methylation of histone H3 via association with histone methyltransferase ‘SETDB1’ [57]. In addition, HBx binds the DNA damage-binding protein 1 (DDB1), which in concert with cullin 4 (Cul4) is part of the E3 ubiquitinase complex [58]. Hereby, it can influence the stability of proteins such as the Smc5/6 (structural maintenance of chromosome proteins 5 and 6) complex, which binds double-stranded DNA and limits HBV transcription [59]. This might constitute an additional direct oncogenic mechanism of HBx because the Smc5/6 complex has been reported to play a role in DNA replication through natural pausing sites and in endogenous DNA damage tolerance [60].

## 7. Genetic risk factors in hepatocellular carcinoma development

Besides the well-known patient-specific risk factors for HCC development in CHB described above, evidence exists for a genetic predisposition due to single-nucleotide polymorphisms (SNPs) [61]. Several SNPs associated with HCC have been reported and expression profiles generated [62,63]. These polymorphisms alter biological pathways, including inflammation, oxidative stress, DNA repair, cell cycle and growth factors [64,65]. The association between aflatoxin B1 and CHB is well established, and a concomitant SNP of GTSM1 (glutathione-*S*-transferase mu1) and GSTT1 (glutathione-*S*-transferase theta1) is associated with a dramatic increase in HCC risk [66]. This indicates that the HCC risk attributable to specific polymorphisms depends on underlying risk factors and specific SNPs are associated with increased HCC risk in CHB. Such polymorphisms include SNPs of MDM2 (mouse double minute 2 homologue) and p53 [67]; XRCC3 (X-ray repair complementing defective repair in Chinese hamster cells 3) [68]; HLA (human leucocyte antigen)-DQ [64]; CTL-4 (cytotoxic T-lymphocyte antigen 4) [69]; GLB1 (galactosidase beta 1) [70] and TGF-β1 (transforming growth factor beta 1) but no other proinflammatory cytokines or interleukin-10 [71]. Nonetheless, these SNPs were mostly detected in collectives of CHB patients from the Far East or Asia and confirmatory studies in other patient populations are required.

In genome-wide association studies of HCV-related HCC, the 5′-flanking region of MICA (MHC class I polypeptide-related sequence A gene) was identified as a susceptibility locus for HCC development, consistent with reduced levels of soluble MICA protein in subjects with the risk allele, supporting an anti-tumour role for this protein [72]. Two further studies identified an SNP in a different gene, DEPDC5 (DEP domain containing 5), associated with HCC risk in Japanese [73] and progression of fibrosis in Europeans [74], although not all studies could confirm this correlation. Thus, additional studies on other populations with stratification of infecting HCV genotype and degree of cirrhosis would provide comprehensive information on the genetic aetiology and heterogeneity of HCV-related HCC. Whole exome sequencing of HCCs of diverse aetiologies has identified driver genes [63,75]; however, no specific virus-induced mutations have been identified to date. Mutations in the telomerase reverse transcriptase promoter are frequently observed in 61% of cirrhotic liver tissue samples, including HCV and HBV infection [76]. Increased TERT activity was observed in HCV core-transfected primary human hepatocytes that show an immortalized phenotype [77]. In line with this observation, somatic mutations in the TERT promoter that enhance TERT expression were shown to be among the earliest and most prevalent neoplastic events in HCC associated with all major aetiologies including HCV [78]. However, further studies are required to validate these observations in different ethnic backgrounds before these host genetic polymorphisms can be used to stratify patients for personalized surveillance or specific targeted therapies. An increased understanding of the genetic and epigenetic changes that drive HCC progression may allow improved therapies in the future; however, the underlying tumour heterogeneity makes such studies challenging.

## 8. A role of hepatitis C virus in promoting a pro-oncogenic microenvironment

HCV is classified into seven genotypes and epidemiological studies show that infection with genotypes 1b and 3 is associated with an increased risk of developing HCC [79,80]. Reports that HCV core gene variants are associated with HCC in patients who have resolved infection [81,82] suggest that viral factors influence progressive liver disease. CHC is often associated with insulin-resistance [83], and the core protein has been shown to dysregulate glucose homeostasis, leading to intrahepatic lipid accumulation and steatosis [84,85]. A recent study highlighted a new role for core to induce mitochondrial damage by impairing mitophagy [86]; the resulting oxidative stress is regarded as a key trigger of HCC initiation and development. *In vivo* studies with HCV core transgenic mice confirmed an imbalance of oxidant/antioxidant state in the liver-induced HCC [87].

HCC exhibits a high degree of genetic heterogeneity indicative of reduced genomic stability [88], and HCV induction of ROS is likely to prime DNA damage. Several studies report that HCV core or NS5A proteins increase ROS and promote oxidative stress in both mouse models and *in vitro* culture systems [89–91]. Further studies report that HCV infection reduces host cells' ability to detect and repair damaged DNA via perturbation of ATM kinase [92–94]. The physiological relevance of these studies is difficult to prove where low-level expression of viral proteins in the infected liver precludes confirmatory studies.

HCC is associated with the development of multifocal, genetically distinct tumours that are suggestive of a field defect affecting the entire liver; however, the nature of the founder cell is poorly understood. An interesting question is whether HCV can replicate in abnormal hepatocytes and act cooperatively with mutations that arise early in the progression to cancer. Harouaka *et al.* [95] reported reduced levels of HCV RNA in HCC compared with adjacent non-tumour tissue and observed increased viral genetic diversity in livers with HCC, supporting a model where HCV replication in the tumour is restricted and compartmentalized. By contrast, Hedegaard *et al.* [12] reported limited evidence of HCV intrahepatic compartmentalization in end-stage liver disease using ultra-deep sequencing technology. These studies highlight the need for further investigation into the relationship between viral diversity, host immune response and ‘phylogeography’ of the liver.

A common feature of oncogenic viruses is their ability to increase cell proliferation via inactivation of host tumour suppressors such as the retinoblastoma (Rb) protein, which represses E2F transcription factors necessary for S-phase entry into cell cycle. HCV-encoded polymerase NS5B has been reported to bind Rb, induces its degradation via host ubiquitin ligase E6AP [96,97] and promotes host cellular proliferation. A recent study showed that NS5B promotes the degradation of NORE1A tumour suppressor [98], an essential factor in HCV replication, highlighting the complexity of viral–host cell interactions. The p53 protein is a critical tumour suppressor which coordinates cell cycle arrest and apoptotic response to DNA damage and other stresses, and p53 mutations are frequently observed in HCC [99]. A number of reports show that HCV proteins core, NS3 and NS5A can associate with p53 [100]; however, the functional consequences of these interactions for p53 activity are complicated by the observation that the most permissive target cell for HCV replication used in these studies expresses a mutated inactive p53 [101]. A recent study reported that HCV induced caspase-3-mediated apoptosis via activation of NLRP3 inflammasome in infected cells and pyroptosis in both infected and non-infected cells, providing a new pathway for HCV to induce hepatocellular damage in both infected and uninfected bystander cells [102]. Despite the many potentially oncogenic features of HCV infection discussed, it is important to note that in the absence of cirrhosis, HCC rarely occurs in CHC. In advanced fibrosis or cirrhosis, HCV genotype 3 infection and insulin-resistance remain important determinants to increase HCC risk even after elimination of virus by antiviral treatment (see below).

## 9. Indirect effects of hepatitis C virus-induced inflammation

HCV infection is sensed by host pathogen-associated molecular pattern receptors that induce interferons (IFNs) and local inflammatory responses. HCV has evolved diverse mechanisms to antagonize these early host immune responses [103]. The majority of infected individuals develop chronic immune-mediated inflammation, accompanied by repeated cycles of hepatocyte destruction and regeneration that are considered to be key drivers in liver cancer. Activated inflammatory cells release ROS and induce lipid peroxidation, which promotes a pro-carcinogeneic environment [104]. Indeed, the observation that most HCV-associated HCC develops in a background of advanced fibrosis and cirrhosis supports a role for host inflammatory responses in this cancer.

Discovering algorithms to identify patients who will develop HCC will increase our understanding to treat and prevent HCC progression. A recent transcriptome meta-analysis including more than 500 cirrhotic human livers demonstrated global regulatory gene modules driving HCC risk and identified the lysophosphatidic acid (LPA) pathway as a central chemoprevention target [105]. LPA is a pleiotropic lipid molecule with potent effects on cell growth and motility, and emerging data highlight an important role in lymphocyte homing and inflammation [106]. Pharmacological inhibition of LPA signalling reduced tumour growth. An independent study confirmed that HCV infection increased autotaxin and associated LPA expression and reported a role for LPA to promote HCV replication [107], providing a pathway for HCV to induce proinflammatory signals that may be pro-oncogenic.

One potential mediator of cellular reprogramming is heritable (epigenetic) regulation of transcription, exemplified by DNA methylation. Tumours associated with chronic inflammation frequently show altered patterns of DNA methylation, including HCC [108]. A recent study showed increased DNA methylation of multiple genes in HCV-infected chimeric mice with humanized livers that were dependent on NK cell activity, demonstrating a role for viral-induced immune responses in regulating hepatocellular methylation status [109]. Wijetunga *et al.* [110] reported DNA methylation of enhancers proximal to genes implicated in liver cancer and stem cell development in HCV-associated HCC, highlighting a role for HCV to influence transcription factor binding to cognate sites in the genome. Reports showing that HCV can stabilize hypoxia-inducible factor-1α [111,112], a transcription factor that regulates vascular endothelial growth factor, provides an additional pathway for HCV to dysregulate the hepatocellular transcriptome and induce de-differentiation via regulating the epithelial-to-mesenchymal transition.

## 10. Effects of hepatitis B virus and hepatitis C virus on hepatocellular microRNAs

MicroRNAs (miRs) are small non-coding RNAs that regulate diverse cell functions including cell proliferation, differentiation and apoptosis. Recent reports highlight aberrant expression of miRs in hepatic tissue from subjects with liver disease and HCC [113], and provide exciting possibilities for the discovery of bio-markers for early diagnosis of viral-associated HCC [114,115].

For CHB, aberrant expression of multiple miRs has been reported to be associated with HCC development. MIR196A2 polymorphism was associated with susceptibility to HBV-related HCC in a male Chinese population [116]. HBx expression may negatively interfere with DNA repair and tumour suppressors by altering expression of multiple miRs through upregulation of HBxAg-upregulated gene 11 (URG11). HBx- and URG11-induced upregulation of *miR-148a* has been shown to drive cell cycle progression and cell migration by suppressing phosphatase and tensin homologue, thus increasing AKT (also known as protein kinase B)–mTOR (mammalian target of rapamycin) signalling [117]. Altered *miR-122a* expression inhibits HBV replication, changes the cell cycle by affecting cyclin G1 expression and inhibits expression of p27 [118].

HCV infection regulates expression of several miRs, including *miR-146a-5p* [119], *miR-196a* [120] and *miR-135a-5p* [27], that regulate metabolic pathways and hepatocarcinogenesis. Expression levels of the liver-specific *miR-122* are inversely associated with HCC of non-viral origin and yet are conserved in HCV–HCC [121]. Since miR-122 is a critical host factor required for HCV replication, this supports a model where HCV infection of founder cells may play an important role in the carcinogenesis process.

## 11. Antiviral treatment and risk of hepatocellular carcinoma development

At the present time, there are no therapies to eliminate HBV infection. IFNα can cure CHB in 3–15% of patients, but has severe side effects and is rarely used. Nucleos(t)ide analogues (NAs) inhibit reverse transcription and limit HBV replication in more than 95% of treated patients and reduce liver inflammation, disease progression and HCC risk. However, these drugs have no effect on viral cccDNA or integrated copy numbers [122] and require long-term administration. Current guidelines recommend antiviral treatment only when serum HBV-DNA levels are greater than 10^3^ copies ml^−1^ (i.e. greater than 2000 IU ml^−1^) and significant inflammatory activity indicated by increased aminotransferase activity in blood or advanced fibrosis has been diagnosed.

A systematic review showed that patients with CHB receiving NA therapy had a greater than 50% lower incidence of HCC (2.8% versus 6.4% of treated and untreated patients, respectively) during a 46 (32–108) month period (*p* = 0.003) [123]. The authors concluded that treatment does not eliminate the risk of HCC. In particular, liver cirrhosis, HBeAg negativity at baseline and failure to remain in virological remission were associated with an increased risk of HCC among treated patients [123]. Additional studies confirmed that patients with CHB remain at risk for HCC development [42,124] despite antiviral treatment. HBV integration and clonal hepatocyte proliferation are already observed early during the course of infection [49] and may be a cause of some of the persistent HCC risk following treatment initiation. This can be taken as an argument for earlier treatment than recommended by the current guidelines. To avoid side effects, reduce costs and minimize the risk of selecting resistant viral variants for long-term NA treatment, this would, however, require a curative treatment approach.

Several studies using IFN-based therapies reported that a sustained virological response (SVR), i.e. successful antiviral therapy that eradicates HCV, reduced the risk of HCC independently of fibrosis stage [125,126]. During an average 10-year follow-up, patients with SVR after antiviral treatment developed HCC in 2.5%, and after spontaneous HCV clearance in 1.6%, of cases, which was dependent on fibrosis stage [27]. A recent multicentre study reported the risk of HCC development in patients with liver cirrhosis to be 1% annually after SVR [127]. In a prospective study of HCV-infected patients with cirrhosis in France for an average of 51 months, a non-SVR was a major determinant of HCC occurrence after the age of 50, with a contribution of past alcohol intake, low platelet count and increased γ-glutamyl transpeptidase [128].

Treatment options for HCV have changed dramatically over the past 5 years with the approval of nucleotide NS5B polymerase inhibitor sofosbuvir in 2013, which showed SVR rates greater than 95% in combination with IFN. Since then, new direct-acting antiviral (DAA) therapies have become available with markedly fewer side effects [74], showing outstanding SVR rates of greater than 90% after eight to 12 weeks of treatment for almost all HCV genotypes. Recent studies have data steered a discussion about a potentially higher recurrence of prior, but successfully treated HCC after DAA therapy and SVR compared with historical IFN-treatment controls [129]. Data on HCC risk after DAA-based SVR only exist as a retrospective or observational study with 1-year follow-up. These studies report an annual HCC incidence rate of 3–5% following successful DAA therapy [74,130], which is higher than previous reports for patients on IFN therapy-based SVR [131]. However, these studies lack control groups, which makes the recent reports on DAA-based SVR and HCC risk hard to interpret. A French prospective cohort study showed lack of evidence of an effect of DAAs on the recurrence of HCC (80% cirrhosis, 189 patients achieving DAA-based SVR, approximately 12% HCC recurrence rate after 20 month follow-up) [132]. Randomized controlled trials will be needed to shed light on the current ongoing debate and may answer the potential role of the drop of HCV-specific immune response after DAA-induced SVR in regard to potentially increased risk of outgrowth of transformed cells and HCC reoccurrence/development.

It has, however, become clear that even the successful DAA therapies for CHC will not be able to eliminate the risk of HCC once high-grade fibrosis or cirrhosis has developed. DAA- and IFN-based regimens showed a considerably reduced, but still remaining risk (0.33%/year) for HCC after HCV cure and highlight the importance of surveillance once liver cirrhosis has developed irrespective of therapy responses [133]. Nevertheless, in countries in which the new DAA therapies are accessible, high rates of SVR and eradication of HCV will have a huge impact on cirrhosis and also HCC incidence in the coming decade.

## 12. Importance of surveillance

The poor prognostic outcome following late diagnosis of HCC, limited curative treatments and prolonged subclinical period of HCC highlight the urgent need for early diagnosis. At the present time, only 30–40% of patients are being diagnosed at early stages (BCLC 0/A). Stratification of patients at-risk and early diagnosis of HCC should be a main objective for forthcoming research. Cofactors such as age greater than 40 years, male gender, duration of infection, alcohol consumption, cigarette smoking, co-infection with both HBV and HCV, HDV or HIV, exposure to aflatoxin B1 and in particular the metabolic syndrome as an emerging cofactor should be taken into account for stratifying patients who need close monitoring because they are at high risk of developing HCC [11,27,35]. Surveillance can reduce mortality by up to 37% using ultrasonography and α-fetoprotein serum levels [134] and application of risk scores for stratifying patient cohorts [135].

HCC can be diagnosed by magnetic resonance imaging, computed tomography and ultrasonography. If there is not typical appearance, biopsy might be required for diagnosis. However, early HCCs are difficult to distinguish from dysplastic nodules [136]. Guidelines recommend surveillance every six months for at-risk populations [24,25], which accommodates the median tumour doubling time [137]. However, there is controversy over the use of serum markers such as α-fetoprotein as surveillance tools for early detection of HCC [27].

Stratification of patients at high risk and implementation of surveillance programmes is needed to detect HCC early. Patients with high-grade fibrosis or cirrhosis, despite successful DAA therapy and HCV cure, require surveillance, given the substantial remaining risk, with old age, diabetes and genotype 3 being independent risk factors [133]. As CHB can lead to HCC development in the absence of cirrhosis in 0.1% per year, surveillance is mandatory. Guidelines may need to be revised because family history of HCC and metabolic syndrome are risk factors for HCC development in the absence of cirrhosis [37].

## 13. Conclusion

Since 2000, the death toll from viral hepatitis has been constantly increasing now exceeding that of HIV infection and malaria. This rise is mainly due to HCC developing on the basis of chronic infection. HBV infection is the major single cause of HCC despite the availability of a vaccine. Therefore, WHO has called for greater efforts to increase global hepatitis B vaccination. Although global vaccination is essential, its impact is limited because the majority of CHB results from mother-to-child transmission hard to prevent. Although the risk of HCC development can be reduced by available antiviral therapy for HBV, it remains significant because the virus has particular features driving hepatocarcinogenesis. This calls for a curative treatment to complement vaccination efforts.

The currently available, highly efficient therapeutic combinations for all HCV genotypes are able to cure CHC and reduce the risk of HCC development, because HCV-associated HCC mainly occurs once liver cirrhosis has developed. These therapies need to become affordable and accessible for the majority of infected individuals. Patients who have progressed to liver cirrhosis remain at risk of developing HCC despite successful antiviral treatment. Thus, broad access to therapeutic intervention before late-stage liver disease has developed as well as surveillance even after successful therapy is required to reduce the death toll from HCC. In addition, a prophylactic vaccine is urgently needed to reduce new infections and to prevent reinfection after antiviral therapy.

## Data accessibility

This article has no additional data.
