# Correction to ‘Viral hepatitis and liver cancer’

**DOI:** 10.1098/rstb.2017.0339

**Published:** 2017-11-20

**Authors:** Marc Ringelhan, Jane A. McKeating, Ulrike Protzer

*Phil. Trans. R. Soc. B* **372**, 20160274 (Published Online 11 September 2017) (doi:10.1098/rstb.2016.0274)

The name of the first author, Marc Ringelhan, was spelt incorrectly as ‘Marc Ringehan’. Therefore, the author list should appear as follows:

Marc Ringelhan, Jane A. McKeating and Ulrike Protzer

